# Amino acid substitutions in hemagglutinin of the 2009 pandemic influenza A(H1N1) viruses that might affect the viral antigenicity

**DOI:** 10.1186/1756-0500-7-951

**Published:** 2014-12-23

**Authors:** Nathamon Kosoltanapiwat, Usa Boonyuen, Phisanu Pooruk, Sopon Iamsirithaworn, Anek Mungaomklang, Kulkanya Chokephaibulkit, Prasert Auewarakul, Pilaipan Puthavathana

**Affiliations:** Department of Microbiology and Immunology, Faculty of Tropical Medicine, Mahidol University, Bangkok, 10400 Thailand; Center for Emerging and Neglected Infectious Diseases, Mahidol University, Bangkok, 10700 Thailand; Department of Molecular Tropical Medicine and Genetics, Faculty of Tropical Medicine, Mahidol University, Bangkok, 10400 Thailand; Department of Microbiology, Faculty of Medicine Siriraj Hospital, Mahidol University, Bangkok, 10700 Thailand; Department of Disease Control, Ministry of Public Health, Nonthaburi, 11000 Thailand; Thepparat-Nakhonratchasima Hospital, Nakhonratchasima, 30280 Thailand; Department of Pediatrics, Faculty of Medicine Siriraj Hospital, Mahidol University, Bangkok, 10700 Thailand

**Keywords:** 2009 pandemic influenza A(H1N1) virus, Hemagglutinin, Hemagglutination inhibition assay, Nucleotide sequencing, Thailand

## Abstract

**Background:**

During 2009 to 2012, Thailand had encountered 4 distinctive waves of the 2009 pandemic influenza A(H1N1) (H1N1pdm) outbreaks. Considering the RNA nature of the influenza viral genome, a mutation in hemagglutinin (HA) gene which led to change in antigenicity of the strains circulating during those epidemic periods is anticipated. It is also uncertain whether the A/California/07/2009 (H1N1) (CA/07) vaccine strain still confers protective immunity against those evolved viruses, the causative agents of the later epidemic waves.

**Methods:**

HA gene segments of 10 H1N1pdm isolates obtained during 2009 to 2012 were sequenced and phylogenetically analysed using ClustalW and MEGA5 programs. A total of 124 convalescent serum samples collected from patients naturally infected during 3 epidemic waves were employed as tools to investigate for antigenic change in HA of these 10 circulating H1N1pdm viruses by hemagglutination inhibition (HI) assay.

**Results:**

A phylogenetic analysis showed that the 10 virus isolates were grouped into 4 clusters corresponding to the time of 4 consecutive outbreaks. An accumulation of amino acid substitutions in HA was observed in viruses derived from the late epidemic waves. Significantly lower antibody titers were observed when CA/07 was tested against convalescent sera collected from the 3 waves (*p* < 0.05) compared to most of Thai isolates; and significantly lower antibody titers were also obtained when virus isolates, retrieved from the third epidemic wave were tested against convalescent sera collected during the first and second wave. These results were suggestive of change in antigenicity of the evolved viruses. Our results also showed some mutation position residing outside the previously reported antigenic site that may involve in an alteration of the viral antigenicity.

**Conclusions:**

Our study demonstrated that convalescent sera collected from individuals naturally infected with H1N1pdm virus were successfully used to reveal a statistically significant change in antibody titers against the currently evolved H1N1pdm viruses as determined by HI assay. Nevertheless, the antibody titers of individual serum against various viruses were less than 4-folded difference as compared to that against the CA/07 vaccine strain. Therefore, CA/07 is still a potent vaccine strain for those evolved H1N1pdm viruses.

## Background

In mid-April 2009, the emergence of a novel influenza A virus was first noticed in Mexico [1]. The virus is a quadruple reassortant, in which the RNA genome is originated from swine, avian, and human influenza viruses [2, 3]. The virus capable of infecting humans, at the time of its emergence, was antigenically new to the world’s population and subsequently spread and caused uncontained influenza outbreaks among humans worldwide. The World Health Organization (WHO) declared the influenza pandemic period between June 2009 and August 2010 [4, 5]. It involved over 18,449 laboratory-confirmed deaths reported to WHO [6], which probably under-represents the total number. On the other hand, an indirect estimation using statistical modeling suggested 201,200 respiratory deaths associated with the H1N1pdm during the first year of the virus circulation [7].

Thailand was among the firstly countries in Southeast Asia that was attacked by the 2009 pandemic influenza. The first documented case was recorded in May 2009 [8], followed by three subsequent dominant waves of the epidemics which lasted for 18 months. The Bureau of Epidemiology, Department of Disease Control, Ministry of Public Health, reported the first wave between May-October 2009, followed by the second in November 2009-April 2010, and the third in May-October 2010. Thailand was heavily attacked by H1N1pdm before the WHO announcement of the pandemic phase. In addition, the actual subsidence of the third wave in Thailand was noted some time after the WHO had announced the end of the pandemic. In January-June 2010, a monovalent pandemic H1N1 vaccine derived from A/California/07/2009 (H1N1) virus (CA/07) was administered to groups of people at risk in Thailand, including healthcare workers [8]. Since then, the H1N1pdm virus has replaced the previous A(H1N1) virus and become the circulating strain in Thailand and worldwide. At present, CA/07 remains a component of the trivalent seasonal influenza vaccine together with influenza A(H3N2) and influenza B strains [9].

Considering the RNA nature of the influenza viral genome, a high rate of mutation resulting in antigenic drift is anticipated due to the lack of the proof-reading capacity of viral RNA polymerase. Influenza hemagglutinin (HA), the surface glycoprotein of a virion, comprises HA1 and HA2 domains. HA1 domain is highly variable; while HA2 is more conserved. The HA1 domain constitutes the HA globular head, which consists of Sa, Sb, Ca, and Cb antigenic sites [10]. HA1 binds sialic acid receptor on the host cytoplasmic membrane, while HA2 mediates fusion of the viral envelope to the endosomal membrane. Thus, the HA molecule determines host cell specificity and induces a protective antibody response [11, 12]. Mutation in the HA gene sometimes results in the HA antigenic change and may result in changing the strain components of a seasonal trivalent vaccine. The hemagglutination inhibition (HI) assay is used to measure HA antibody titer for serodiagnosis or vaccine evaluation. HI antibody titers equal or greater than 40, either as the result of natural infection or vaccination, are considered immune to re-infection or protective against infection by homologous influenza strains [13]. During the post-pandemic period, the World Health Organization has recommended monitoring of the virus for important genetic and antigenic changes [14]. Knowing the changes will be useful for prediction of potential future outbreaks, and maintaining a matching between circulating influenza strains and vaccine strains.

In this study, 10 H1N1pdm strains isolated from Thai patients during the 4 epidemic waves (2009-2012) were analysed for genetic and antigenic variation in comparison with the CA/07 vaccine strain. A phylogenetic tree was constructed from their HA gene sequences. These 10 virus isolates were allowed to react with convalescent sera collected from patients infected during the first 3 epidemic waves; and a possible change in the virus antigenicity was analysed based on the levels of the HI antibody titers obtained. Although a panel of immune ferret sera immunized with particular strains is usually employed for determining the change in virus antigenicity by HI assay, our study suggested that convalescent sera collected during different epidemic waves from individuals naturally infected with H1N1pdm virus might serve as an alternative tool to reveal an antigenic variation of the evolved viruses. The 3-dimensional structure of the viral HA, with mutation, was also predicted by homology modeling.

## Results

### Analysis of the HA sequences of the Thailand isolates

**Figure 1:**
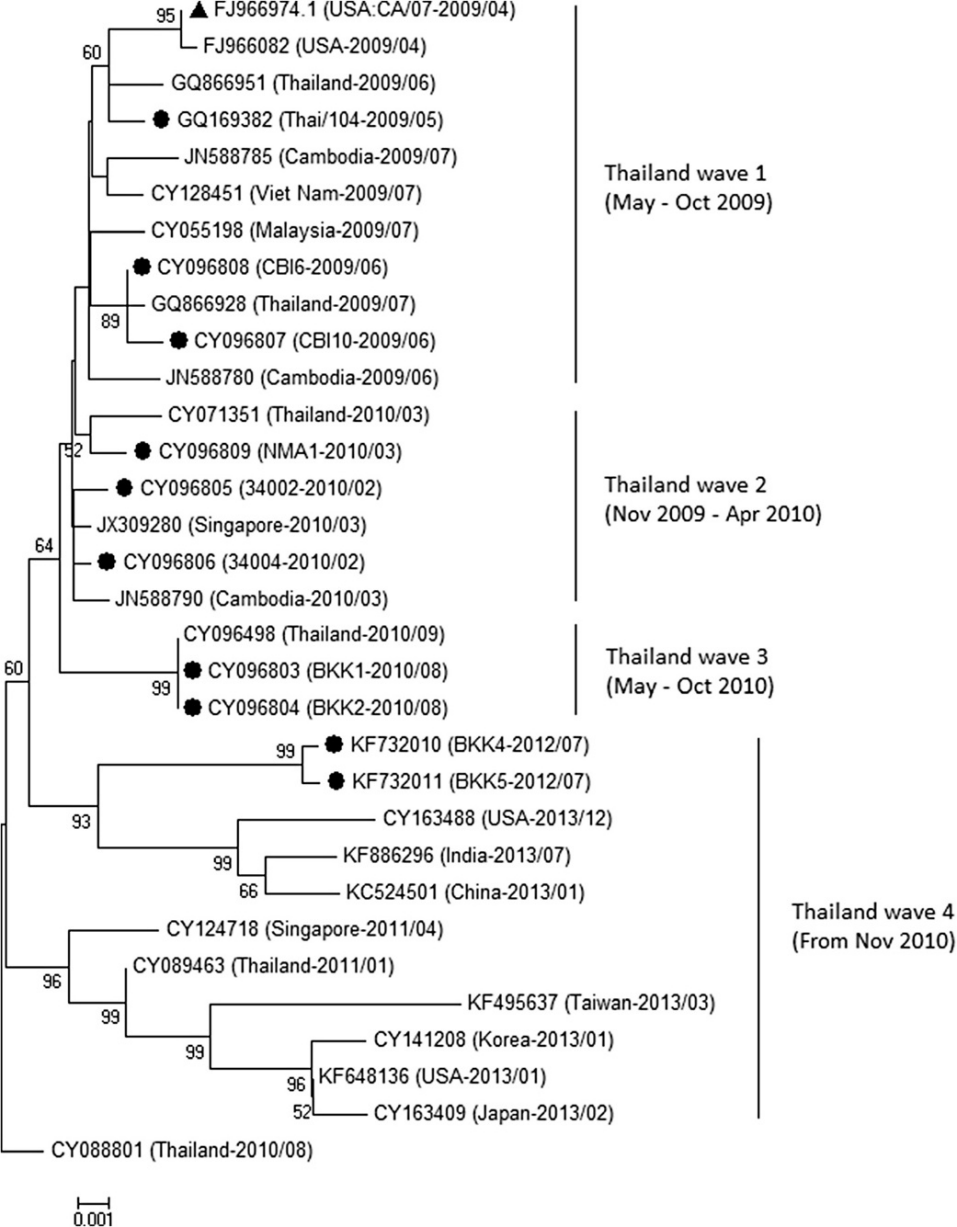
**Phylogenetic analysis of HA gene of the 2009 pandemic influenza A(H1N1) viruses.** The HA gene sequences of 10 H1N1pdm Thai isolates, vaccine strain and other viruses retrieved from the NCBI Influenza Virus Resource database were analysed with sequence CY088801 (Thailand-2010/08) as a root of the phylogenetic tree. The GenBank accession numbers, country in which the viruses were detected, and the year and month of detection, are shown. Bootstrap values of more than 50 are indicated on each branch. Circle, strains isolated in this study; triangle, vaccine strain.

Detailed information of the 10 H1N1pdm isolates used in this study is shown in Table 1. Eight strains were isolated during the three epidemic waves which lasted from May 2009 to August 2010; and two strains were isolated during post-pandemic period in July 2012 (defined here as the fourth wave of the Thailand epidemic). Full-length HA genes of the study viruses were analyzed against CA/07 (vaccine strain) and other 21 H1N1pdm strains isolated in Thailand and neighboring countries during the years 2009-2013, using sequencing data retrieved from the NCBI influenza database (http://www.ncbi.nlm.nih.gov/genomes/FLU/). Multiple sequence alignment showed >97% identity to the HA genes of the 32 viruses analyzed. The Thailand viruses isolated from each epidemic wave were accordingly clustered together, corresponding with the times of epidemics (Figure 1). Although the viruses from wave 1 and wave 2 were not separated into distinctive clusters, a clear separation could be observed with the viruses from wave 3 and wave 4.

**Table 1:** **The 2009 pandemic influenza A(H1N1) viruses used for phylogenetic analysis and HI assay**

Virus	Month of specimen collection (yyyy/mm)	Wave no.	Place of specimen collection	Passage history	Abbreviation	Accession no.
A/California/07/2009	-	Vac	-	E4 MD5	CA/07	FJ966974.1
A/Thailand/104/2009	2009/05	1	Mexico imported case	MD5	Thai/104	GQ169382
A/Thailand/ICRC-CBI6/2009	2009/06	1	Chonburi, Thailand	MD5	CBI6	CY096808
A/Thailand/ICRC-CBI10/2009	2009/06	1	Chonburi, Thailand	MD5	CBI10	CY096807
A/Thailand/ICRC-BKK34002/2010	2010/02	2	Bangkok, Thailand	MD5	34002	CY096805
A/Thailand/ICRC-BKK34004/2010	2010/02	2	Bangkok, Thailand	MD4	34004	CY096806
A/Thailand/ICRC-NMA1/2010	2010/03	2	Nakhon Ratchasima, Thailand	MD5	NMA1	CY096809
A/Thailand/ICRC-BKK1/2010	2010/08	3	Bangkok, Thailand	MD5	BKK1	CY096803
A/Thailand/ICRC-BKK2/2010	2010/08	3	Bangkok, Thailand	MD5	BKK2	CY096804
A/Thailand/ICRC-BKK4/2012	2012/07	4	Bangkok, Thailand	MD6	BKK4	KF732010
A/Thailand/ICRC-BKK5/2012	2012/07	4	Bangkok, Thailand	MD5	BKK5	KF732011

Vac; vaccine strain.

Amino acid sequences of HA protein, including the HA1 globular head (amino acid positions 42-275 which spanned the Sa, Sb, Ca, and Cb antigenic sites) and the HA2 domain of the study viruses were analyzed. The amino acid changes in those regions were compared with those of the vaccine strain, CA/07, as the reference sequence (Table 2). The result showed a higher number of amino acid substitutions in the viruses collected from the late epidemic waves than those from the early epidemic waves. As expected, mutations were more common in the HA1 domain than the HA2 domain.

**Figure 2:**
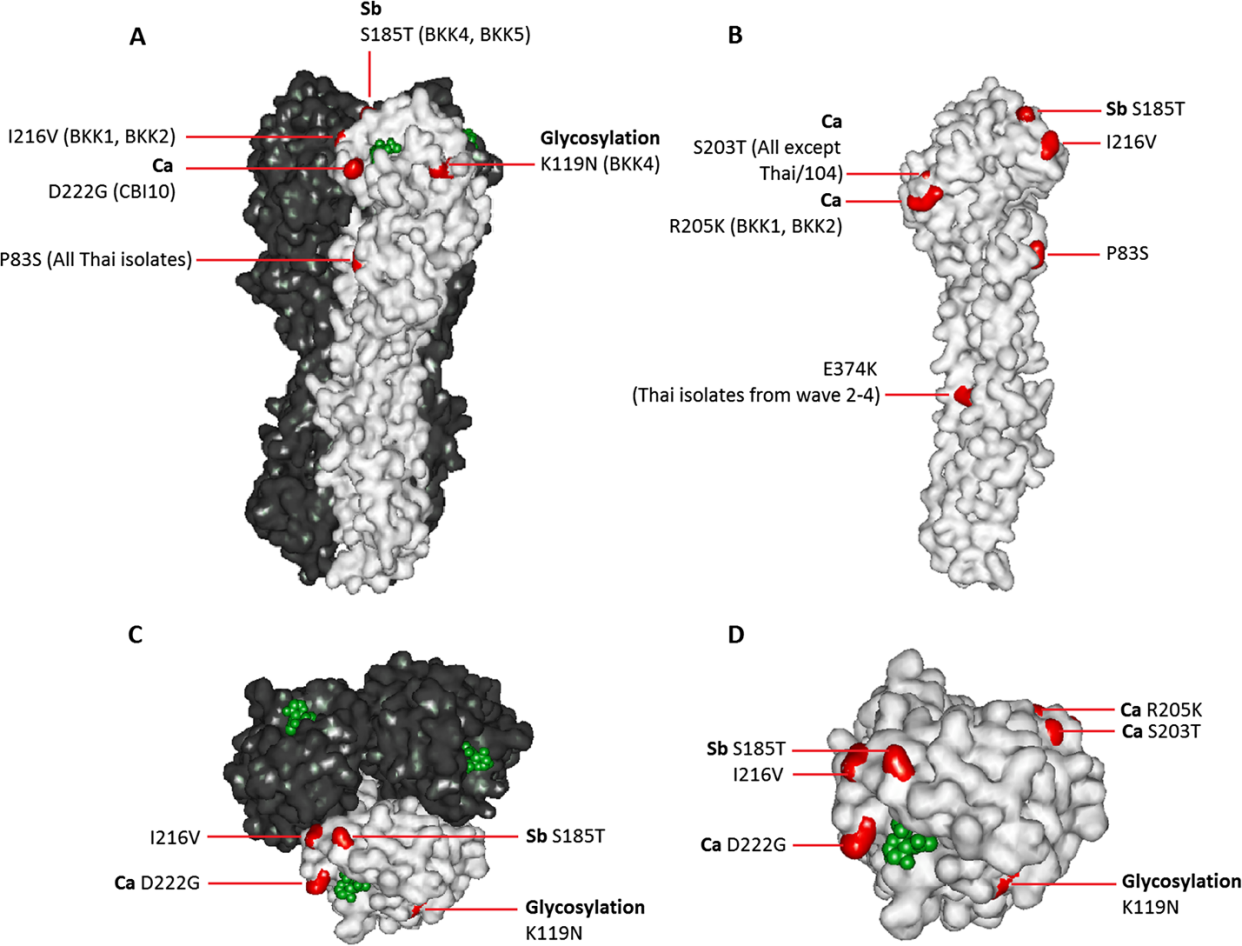
**Surface representation of HA protein.** Amino acids associated with antigenic sites (Sb and Ca) and N-glycosylation are highlighted in orange in **(A)** side view of HA trimers, **(B)** side view of HA monomer, **(C)** top view of HA trimers and **(D)** top view of HA monomer. Sialic acid is shown in green. Figures were generated by Discovery Studio Visualizer-Accelrys.

Considering the antigenic site, Sa, Sb, Ca, and Cb on the HA1 globular head, no difference was found in the amino acid compositions between Thai/104 (the imported virus from Mexico) and CA/07. However, amino acid substitution S203T on Ca was noted in all other Thai strains, while the mutation in Sb was detected in viruses isolated during the fourth epidemic wave. Surprisingly, no amino acid change was observed in the Sa and Cb sites. In addition, the BKK4 strain from the fourth wave contained amino acid substitution K119N, which contributes to the acquisition of a new N-linked glycosylation site. Therefore, it is predicted that BKK4 contains 7 glycosylation sites on the HA1 globular head instead of 6 sites as found in the other viruses. Amino acid substitutions that may affect antigenicity and N-linked glycosylation sites are demonstrated in the homology model of HA protein in Figure 2.

**Table 2:** **Amino acid similarity among the HA proteins of different H1N1pdm isolates, compared with A/California/07/2009**

Region	Number of amino acids involved (position)	Number of amino acid residues identical to A/CA/07/2009 (H1N1) (amino acid substitution position)									
		Thai/104	CBI6	CBI10	34002	34004	NMA1	BKK1	BKK2	BKK4	BKK5
HA1 globular head	234 (42-275)	233 (P83S)	232 (P83S)	230 (P83S) (K119E)	231 (P83S) (S128L)	232 (P83S)	232 (P83S)	228 (P83S) (D97N) (I216V) (V249L)	228 (P83S) (D97N) (I216V) (V249L)	227 (P83S) (D97N) (K119N)* (V249L)	227 (P83S) (D97N) (N129D) (V249L)
Sa	13	13	13	13	13	13	13	13	13	13	13
Sb	12	12	12	12	12	12	12	12	12	11 (S185T)	11 (S185T)
Ca	19	19	18 (S203T)	17 (S203T) (D222G)	18 (S203T)	18 (S203T)	18 (S203T)	17 (S203T) (R205K)	17 (S203T) (R205K)	17 (H138R) (S203T)	17 (H138R) (S203T)
Cb	6	6	6	6	6	6	6	6	6	6	6
HA2	222 (328-549)	221 (I404M)	222	222	221 (E374K)	221 (E374K)	221 (E374K)	221 (E374K)	221 (E374K)	220 (E374K) (S451N)	220 (E374K) (S451N)

17 amino acids of the signal peptide starting from M are not included. The first HA position starts from D.

Amino acid residues in Sa, Sb, Ca, and Cb sites of the globular head are defined according to reference [10].

*Substitution point affecting glycosylation.

### HI assay of sera from the H1N1pdm patients against various virus isolates

**Figure 3:**
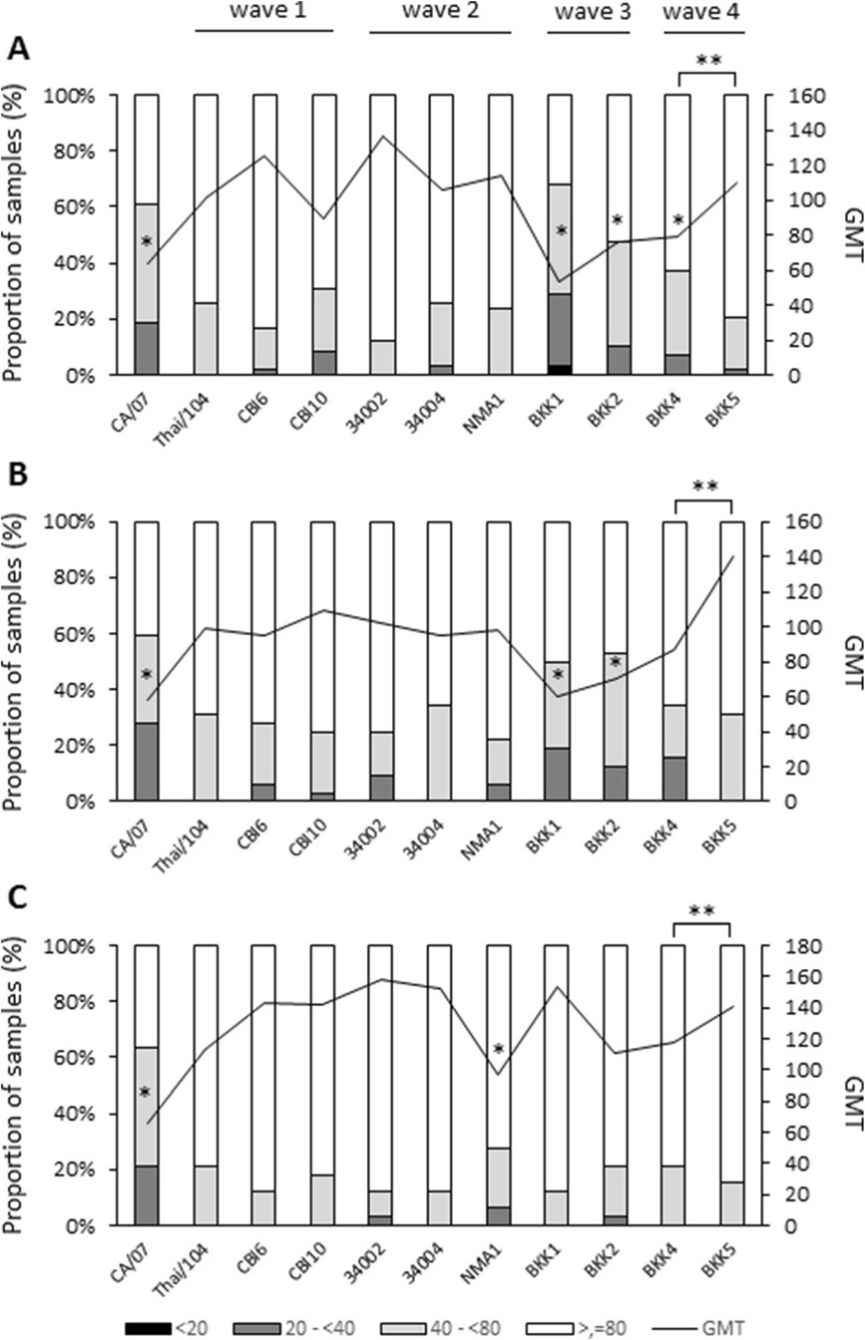
**Antibody titers against the 2009 pandemic influenza A(H1N1) viruses.** The H1N1pdm antibody positive convalescent sera collected during Thailand’s 2009 pandemic influenza outbreaks, wave 1 **(A)**, wave 2 **(B)**, and wave 3 **(C)**, were tested against 11 isolates of 2009 pdmH1N1 viruses by HI assay. Stacked bars show the proportion of samples with different HI titers, while the line denotes the geometric mean titer (GMT). *represents a statistically lower GMT compared with the GMT of viruses isolated from the same epidemic wave as sera, **represents a statistical difference between the GMT of BKK4 and BKK5, *p* < 0.05, tested by Wilcoxon Signed Ranks Test.

In this study, convalescent sera collected from laboratory-confirmed cases of H1N1pdm infection during the three epidemic waves were tested by HI assay against 11 virus isolates, including CA/07 virus and 10 Thailand isolates originating from the four epidemic waves. Using the CA/07 virus as the test antigen, the HI antibody titers ≥40 were found in 81.4% (48 of 59), 71.9% (23 of 32), and 78.8% (26 of 33) of the convalescent serum samples collected from the first, second and third epidemic waves, respectively. Variation in the number of sera with HI antibody titers ≥40 was found when these panels of sera were assayed against the Thailand viruses belonging to different epidemic waves (Table 3). The geometric mean titer (GMT) of HI antibody in sera collected from the 3 epidemic waves, as assayed against CA/07 and 10 Thailand isolates are shown in Figure 3. The GMT of sera from the first and second epidemic waves against CA/07 virus was significantly lower than GMTs against the Thailand viruses originating from the first and second epidemic waves (*p* < 0.05), but not against viruses from the third wave, which contained a higher number of amino acid substitution positions. Regarding serum samples from the third epidemic wave, the GMT against all viruses were higher than CA/07. Furthermore, BKK4 virus of the fourth wave showed a significantly lower GMT compared with the BKK5 virus of the same wave, when reacted with sera from all 3 waves.

**Table 3:** **Numbers of sera with HI titer ≥40 when tested against different viruses**

Virus		% with HI titer ≥40		
		Serum wave 1 (n = 59)	Serum wave 2 (n = 32)	Serum wave 3 (n = 33)
	**CA/07**	**81.4 (48/59)**	**71.9 (23/32)**	**78.8 (26/33)**
Thailand wave 1	Thai/104	100 (59/59)	100 (32/32)	100 (33/33)
	CBI6	98.3 (58/59)	93.8 (30/32)	100 (33/33)
	CBI10	91.5 (54/59)	96.9 (31/32)	100 (33/33)
Thailand wave 2	34002	100 (59/59)	90.6 (29/32)	97 (32/33)
	34004	96.9 (57/59)	100 (32/32)	100 (33/33)
	NMA1	100 (59/59)	93.8 (30/32)	93.3 (31/33)
Thailand wave 3	BKK1	71.2 (42/59)	81.3 (26/32)	100 (33/33)
	BKK2	89.8 (53/59)	87.5 (28/32)	97 (32/33)
Thailand wave 4	BKK4	93.2 (55/59)	84.4 (27/32)	100 (33/33)
	BKK5	98.3 (58/59)	100 (32/32)	100 (33/33)

## Discussion

HA is a viral protein that induces protective antibody response. With a pressure from neutralizing antibodies generated upon natural infection or vaccination, changes in the HA antigenic determinant are driven in order that the virus can evade the host immune response [15]. It has been demonstrated that the percentages of amino acid divergence in HA are highest, when compared with other genome segments [16]. In this study, HA sequences of 10 Thailand H1N1pdm viruses isolated in 2009-2012 were analysed genetically against sequencing data from the NCBI Influenza Virus Resource database. Phylogenetic analysis showed that the HA genes of our isolates formed 4 clusters, corresponding to the time of virus isolation, i.e., the first, second, third and fourth epidemic waves in Thailand. The accumulation of nucleotide substitutions in the late viruses indicated that the H1N1pdm viruses gradually evolved at the nucleic acid and amino acid levels during the past 5 years.

We sought to elucidate whether changes in the HA gene of our viruses affected the ability of HA protein to react with the anti-HA antibodies naturally raised in Thai patients. Convalescent sera from H1N1pdm patients infected during the 3 epidemic waves were assayed against 10 H1N1pdm viruses derived from the 4 epidemic waves by HI assay, the method with a potential to detect changes on the HA globular head. It was noticed that CA/07, the vaccine strain, yielded a statistically significant lower HI titers than most of the Thailand viruses originating from different epidemic waves; nevertheless, the antibody titer against CA/07 in individual serum was less than 4-folded difference as compared to those Thailand isolates (data not shown). Even though the cross reactive HI titers of ≥ 40 were found in 33% of adults of age more than 60 years prior to H1N1pdm vaccination [17], 2 (6.1%) of 33 of the acute sera from our subjects with unknown age possessed this antibody level (data not shown). Therefore, an issue on cross reactive antibody present in old aged people that might affect our laboratory interpretation is less likely in this study. P83S amino acid substitution in the HA1 globular head has been observed in all Thailand viruses, but not CA/07, suggesting that this point mutation may be associated with an increase in HI titer as investigated with the Thailand viruses. Nevertheless, P83S substitution is not located in the previously reported antigenic sites (Sa, Sb, Ca, and Cb). The sera from all 3 epidemic waves reacted with the viruses of the first and second waves at comparable titers, indicating that viruses of the first and the second waves are not antigenically distinct. This concurs with a report by the Ministry of Public Health, Thailand which showed that the attack rate of H1N1pdm in the second wave was lower than that of the first wave [8]. This could be due to the protective immunity developed in Thai population after the first H1N1pdm epidemic wave. Based on amino acid analysis, the viruses from the third wave which accumulated a high number of amino acid substitutions, showed relatively poor activity when reacted with sera from the first and second waves. Observation of the HA1 globular head has demonstrated amino acid substitutions I216V and R205K in viruses from the third wave only, and this might relate to decreased HI titers when these viruses were used as test antigens. Nevertheless, the substitutions I216V are located outside previously reported antigenic sites; while R205K is located on antigenic site Ca. No substitution was found in the Sa and Cb regions of any virus studied. It is likely that amino acid residues outside the previously reported antigenic sites also play a role as additional antigenic determinants of viruses. Our finding was concordant with that reported by Deem *et al.* [18] who employed an entropy-based method and demonstrated that more amino acid positions outside the previously defined antigenic site of H1 HA may involve in the HA antigenicity.

D97N, S185T, S203T, D222G, and E374K, observed in this study, have been reported in viruses from Thailand and other regions [16, 19, 20]. S203T present in all viruses except Thai/104 and S185T present in the viruses of the fourth wave, were located in the Ca and Sb sites, respectively. However, their involvement in antigenicity changes is uncertain. HA protein modeling suggested that the amino acid position 203 is not located on the surface of HA trimer. D222G, the mutation found in a virus isolate from a dead case (CBI10), is previously shown to be associated with the more virulent phenotype in humans and mice [19, 21, 22]. E374K substitution on HA2 stalk region has been observed in most of the H1N1pdm strains isolated after the year 2010 [23]. This position, in particular, has been found in viruses isolated from vaccinees who received the monovalent 2009 H1N1 influenza vaccine, and from a number of fatal cases [24]. The E374K is suggestive for an increase in a structural and acid stability of the HA trimers involving in membrane fusion, and potentially affects the viral antigenicity and adaptation [23, 25, 26]. Among 10 H1N1pdm viruses in this study, none of our 3 strains in the first epidemic wave contained this E374K substitution, while it was detected in all 7 strains isolated in the later epidemic wave. Four of these 7 strains were isolated from cases of vaccine failure (BKK1, BKK2, BKK4, and BKK5), but no case was fatal. The simultaneous occurrences of E374K with D97N and S203T, and of E374K with S185T among our Thailand viruses were previously observed among the strains isolated from the other geographical regions [19, 27]. K119N, a potential glycosylation site on the HA globular head, was found in BKK4, but not BKK5 virus derived from the fourth epidemic wave. This substitution may be responsible for a decrease in GMT when BKK4 was compared with the BKK5 virus. This K119N glycosylation site is situated close to the ligand binding site; hence, it could mask the receptor binding epitope on the HA surface [10]. It was noted that K119N was found among the candidate vaccine strains with improved virus yields in eggs [28, 29].

## Conclusions

Collectively, our results showed a gradual change in HA proteins of the H1N1pdm viruses isolated in Thailand during 2009-2012. This variation has resulted in the viral antigenic changes as a significant difference in HI antibody titer could be clearly demonstrated when convalescent sera were cross-checked with the vaccine strain and virus isolates derived from various epidemic waves. However, the HI titers found in this study are not more than 4-folded difference, indicating that the current CA/07 vaccine strain should still be effective.

## Methods

### Subjects and samples

A total of 124 archival samples of convalescent sera comprised 59 samples collected from male conscripts in July 2009 during the first epidemic wave, 32 samples (15 male conscripts and 17 general population of age between 2-42 years old) collected between December 2009 and March 2010 during the second epidemic wave, and 33 samples collected from male inmates in September 2010 during the third epidemic wave. These sera had been kept at -20°C until tested. As suggested by occupation, the ages of male conscripts were approximately 21 years old. Unfortunately, ages of some subjects including the male inmates were not known. Infection status of the subjects was confirmed by detection of the viral genome in respiratory samples by real time reverse transcription-polymerase chain reaction (real time RT-PCR) using the protocol established by the U.S. Centers for Disease Control and Prevention (CDC) [30]. Collection of respiratory and blood samples is the standard practice for investigation of influenza outbreak by the Bureau of Epidemiology, Ministry of Public Health. This study which employed these blood samples was approved by Siriraj Institutional Review Board, Faculty of Medicine Siriraj Hospital, Mahidol University, Thailand.

### Viruses

Eleven strains of H1N1pdm, isolated during the years 2009-2012, were employed as test antigens in HI assay. Except for CA/07, which was obtained from the National Institute for Biological Standards and Control (NIBSC), UK, all viruses were isolated in Thailand. The HA gene of these viruses were subjected to nucleotide sequencing, and the complete HA sequences were submitted to the NCBI Influenza Virus Resource database (http://www.ncbi.nlm.nih.gov/genomes/FLU/). Descriptions of the viruses are shown in Table 1. The viruses were propagated in Madin-Darby Canine Kidney (MDCK) cells, obtained from the American Type Culture Collection (CCL-34), maintained in Eagle's minimum essential medium (Invitrogen, Grand Island, NY) supplemented with 2 μg/ml of L-1-tosylamido-2-phenylethyl chloromethyl ketone (TPCK)-treated trypsin (Sigma, St. Louis, MO). The culture supernatant was harvested, centrifuged at 4°C for 15 minutes, aliquoted, and kept frozen as virus stock at -80°C until used.

### Nucleotide sequencing and analysis

Total RNA was extracted from the virus stock by QIAamp® Viral RNA Mini Kit (QIAGEN Inc., Valencia, CA) using the procedure described in the manufacturer’s instructions. The HA gene was amplified by QIAGEN® OneStep RT-PCR kit (QIAGEN Inc.) using 2 overlapping HA primer sets: HA1F; 5’ATACGACTAGCAAAAGCAGGGG3’ and HA943R; 5’GAAAKGGGAGRCTGGTGTTTA3’ (product size of 943 bp) and HA736F; 5’AGRATGRACTATTACTGGAC3’ and HA1778R; 5’GTGTCAGTAGAAACAAGGGTGTTT3’ (product size 1042 bp). The protocol for RT-PCR comprised the steps of reverse transcription at 50°C for 45 min and PCR activation at 95°C for 15 min, followed by 5 cycles of denaturation at 95°C for 30 sec, annealing at 50°C for 30 sec, and extension at 72°C for 1 min, then another 30 cycles of denaturation at 95°C for 30 sec, annealing at 55°C for 30 sec and extension at 72°C for 1 min, with the final extension step at 72°C for 10 min. The PCR products were gel purified and sent to BioDesign Co., Ltd., Thailand for nucleotide sequencing. Nucleotide sequences of the 2 partial HA fragments were assembled using the BioEdit program to yield the full length HA sequence. A phylogenetic tree was constructed using the MEGA 5 program, by applying the neighbor-joining algorithms with Kimura’s two-parameter distance model and 1,000 bootstrap replicates [16, 31]. Prediction of the potential glycosylation site employed NetNGlyc 1.0 (http://www.cbs.dtu.dk/services/NetNGlyc/). The glycosylation site was defined as Asn-X-Ser/Thr, where X represents any amino acid, except proline [10].

### Hemagglutination inhibition (HI) assay

The HI assay was performed as previously described [32, 33]. The test sera were mixed with receptor-destroying enzyme (RDE) (Denka Seiken, Tokyo, Japan) at a serum: RDE ratio of 1: 3, to remove non-specific inhibitor. The mixture was incubated at 37°C in a water bath for 16-18 h, followed by heat inactivation at 56°C for 30 min. The RDE-treated serum was added with 5 volumes of 0.9% NaCl and 1 volume of 50% erythrocyte followed by incubation at 4°C for 1 h, to remove non-specific agglutinator. The reaction tube was spun and the treated serum at a dilution of 1: 10 was obtained. For the HI assay, the test serum was 2-fold serially diluted from a starting dilution of 1: 10 to 1: 1280 in a 25 μl volume, and 25 μl of the virus suspension were added to a final concentration of 4 HA units. After incubation at ambient temperature for 30 min, 50 μl of 0.5% goose erythrocytes were added and the mixture was incubated at 4°C for 30 min. HI antibody titers were determined and defined as the reciprocal of the highest serum dilution that completely inhibited the hemagglutination reaction. Each run included positive control serum and back titration of viral antigen. The experiments were run in duplicate and the results were presented as geometric mean titer (GMT). The difference in HI titers of the test sera as assayed against different virus strains was statistically analyzed by Wilcoxon Signed Ranks Test. The statistical significance was set at *p* < 0.05.

### Homology modeling

A structural model of the influenza HA molecule was constructed using SWISS-MODEL, based on homology modeling using the crystal structure of the HA molecule of H1N1pdm (PDB ID: 3LZG) as template [34]. The model obtained was validated by PROCHECK [35].
